# Seeing Others, Feeling Less Grounded: How Human Presence in Images Reduces Place Attractiveness

**DOI:** 10.3390/bs16030433

**Published:** 2026-03-16

**Authors:** Dengfeng Cui, Xinyi Zhu, Lin Wang, Zengxiang Chen

**Affiliations:** 1 School of Economics and Management, Shihezi University, Shihezi 832000, China; cuidengfeng2025@163.com (D.C.); zhuxinyi20002025@163.com (X.Z.); 2 International School of Business & Finance, Sun Yat-sen University, Zhuhai 519082, China; chzengx@mail.sysu.edu.cn

**Keywords:** groundedness, human presence, place attractiveness, landscape type

## Abstract

People increasingly rely on photographs shared by others when deciding where to travel. Although human presence in such images is prevalent and often assumed to enhance place appeal, little research has examined whether it might also produce unintended drawbacks. Drawing on the theoretical perspective of groundedness, this research proposes that depicting people in place photographs can undermine viewers’ sense of being psychologically anchored to the place, thereby reducing perceived attractiveness. Across one field study and three controlled experiments, we find consistent evidence that human presence lowers place attractiveness by diminishing feelings of groundedness. This effect emerges in cultural but not natural landscapes and cannot be explained by alternative mechanisms such as psychological ownership. By introducing groundedness as a novel psychological mechanism, this research advances understanding of how human presence in imagery shapes individuals’ affective and evaluative responses to destinations.

## 1. Introduction

Visual content has become central to how individuals imagine and choose destinations. Photographs and videos shared by influencers and official tourism organizations now serve as persuasive tools that shape perceptions of attractiveness and influence visit intentions (Z. Xie et al., 2022; K. Zhang et al., 2023). A notable feature of such imagery is the frequent inclusion of people (e.g., for instance, a tourist taking a photo at a popular Instagram spot). Both marketers and travelers often assume that human presence enhances the relatability and social appeal of place images (e.g., Back et al., 2020; Y. W. Li & Wan, 2025). Indeed, approximately 40% of destination photographs include human figures (Deng & Liu, 2021; Nikjoo & Bakhshi, 2019), reflecting the widespread belief that “seeing others” enhances promotional effectiveness. Yet, the question remains: might these seemingly engaging depictions sometimes backfire?

This research argues that the inclusion of people in place photographs can paradoxically diminish perceived place attractiveness by disrupting viewers’ feelings of groundedness—a psychological state reflecting one’s sense of emotional attachment, stability, and belonging to a place (Eichinger et al., 2022; Y. Xie & Desouza, 2025). Groundedness emerges through sustained engagement with a place’s environmental, social, and cultural cues, enabling individuals to internalize its symbolic meanings and imagine themselves as part of it (Bruckberger et al., 2023). Such feelings foster emotional security and continuity, ultimately enhancing tourists’ satisfaction, loyalty, and overall travel experience (Bruckberger et al., 2023; Guo et al., 2024). However, when place imagery features other unfamiliar travelers, these human figures may inadvertently disrupt this process. Because faces and bodies naturally capture visual attention, their presence can divert perceptual and cognitive resources away from place-defining elements, reducing the viewer’s ability to process and internalize the setting’s aesthetic and cultural essence (Wang et al., 2024). Moreover, when the depicted individuals appear socially or culturally incongruent with the viewer’s self-concept, they can elicit a subtle sense of “outsiderness,” further undermining psychological connection to the place (Z. Y. Lu et al., 2024; Reed et al., 2012). Consequently, rather than reinforcing place appeal, human presence can erode the viewer’s groundedness and, in turn, diminish perceived attractiveness.

Despite these insights, a critical tension and theoretical gap remain. Prior literature presents mixed findings on the effect of human presence (Y. W. Li & Wan, 2025; Z. Y. Lu et al., 2024), lacking an integrative framework to explain when and why it can backfire. Specifically, the underlying psychological mechanism that translates visual social cues into diminished place appeal is not well understood, and the contextual boundary conditions for this effect are unclear. To address this gap, the present research introduces the construct of groundedness as a novel mediator. We propose that human presence in images undermines this sense of groundedness, thereby reducing palce attractiveness. Furthermore, we posit that this effect is contingent on landscape type, being salient for cultural landscapes but attenuated for natural landscapes. Thus, this research aims to test two core hypotheses: (H1) Human presence reduces perceived place attractiveness by diminishing feelings of groundedness; and (H2) The negative effect of human presence in image is moderated by landscape type, occurring only for cultural landscapes.

## 2. Theoretical Background

### 2.1. Human Presence in Place Imagery

Prior research has long recognized that the presence of others can shape consumers’ attitudes and behaviors, even in non-interactive contexts (Bandura, 1977; Cialdini & Goldstein, 2004). For instance, merely observing a stranger with a similar body type trying on clothing can influence a consumer’s purchase decisions (Argo et al., 2005). Likewise, simply witnessing residents’ collective activities—such as traditional dance performances—can foster a stronger sense of place attachment by enhancing tourists’ emotional connection and perceived self-efficacy (Richards, 2007). These findings highlight the potential for human presence—whether directly experienced or vicariously observed—to strengthen affective bonds with places.

Building on this insight, recent research has examined the role of human presence in place photographs, a dominant form of visual communication in online tourism marketing (Z. Xie et al., 2022). On one hand, studies show that including people in place imagery can heighten attractiveness by stimulating mental simulation, enabling viewers to imagine themselves within the scene and thereby increasing their intention to visit (Back et al., 2020; F. S. Li et al., 2025; Y. W. Li & Wan, 2025; K. Zhang et al., 2023). On the other hand, emerging evidence suggests that human presence may sometimes undermine place appeal. For travelers seeking contemplative or culturally immersive experiences, visible others can distract attention and disrupt psychological connection with the place. Prior work demonstrates that crowding and social presence redirect visual attention away from environmental features, elevating stress and lowering satisfaction (P. Li et al., 2023; Wang et al., 2024). Similarly, Diehl et al. (2016) show that self-presentational motives triggered by others’ presence can shift individuals from experiential to evaluative mindsets, thereby reducing immersion. More recently, Z. Y. Lu et al. (2024) find that images depicting strangers can decrease liking for a venue by diminishing feelings of psychological ownership.

In summary, existing findings reveal a conceptual tension: while human presence in place photographs can enrich social cues, stimulate imagination, and foster emotional engagement, it may also distract, intrude, or signal exclusivity, thereby diminishing perceived attractiveness (e.g., Y. W. Li & Wan, 2025; F. S. Li et al., 2025; Z. Y. Lu et al., 2024). This lack of consensus underscores the need for a more nuanced theoretical framework explaining when and why human presence in place imagery enhances or undermines viewers’ evaluations. To address this gap, the present research introduces the construct of groundedness as a novel lens for understanding the psychological processes that govern responses to human presence in place photographs.

### 2.2. Groundedness

Groundedness refers to a sense of belonging and emotional attachment that individuals develop through the integration of environmental features, social interactions, and cultural experiences (Eichinger et al., 2022; Y. Xie & Desouza, 2025). It encompasses both cognitive and affective dimensions: physical attributes (e.g., landscapes, architecture) provide the perceptual foundation for connection (McAndrew, 1998), while social exchanges and cultural practices deepen an individual’s psychological attachment to a place (Thompson & Coskuner-Balli, 2007). When people feel “anchored” in an environment, groundedness affords emotional stability and a sense of security, thereby enhancing their evaluation of the destination (Bruckberger et al., 2023; Guo et al., 2024).

Within consumer behavior and marketing research, groundedness is typically treated as an affective psychological state characterized by feelings of being “rooted” in a particular place or context (Eichinger et al., 2022). Rather than emerging solely from direct, prolonged engagement, such feelings can also be evoked indirectly through symbolic cues embedded in products, experiences, or mediated representations. Prior studies show, for example, that observing artisans engaged in traditional craftsmanship can enhance consumers’ appreciation of local products by reinforcing perceived cultural embeddedness (Fuchs et al., 2015). Similarly, visual media—especially photography—can convey place-based meanings that allow viewers to vicariously experience connection, continuity, and belonging, even in the absence of physical presence (Van Osselaer et al., 2020). These findings suggest that groundedness is not only shaped by lived experience but is also highly sensitive to how places are visually represented.

Importantly, groundedness has been shown to yield a range of desirable outcomes, including stronger preferences, greater willingness to pay, and enhanced subjective well-being, as it instills feelings of strength, stability, and safety (Eichinger et al., 2022). In tourism contexts, groundedness serves as a critical affective foundation for tourist satisfaction, loyalty, and meaningful place experiences (Bruckberger et al., 2023; Guo et al., 2024). Because such feelings rely on viewers’ ability to process and internalize place-defining cues, the visual composition of place imagery becomes particularly consequential. Visual elements can either facilitate sustained engagement with environmental and cultural features or divert attention toward competing stimuli. One such element is the inclusion of human figures, which may subtly alter attentional focus and interpretive processes, thereby shaping how viewers experience groundedness when encountering place imagery.

### 2.3. Landscape Type

Landscape types are commonly distinguished between cultural landscapes and natural landscapes, which differ fundamentally in their underlying logic of value creation and core experiential essence for visitors (F. S. Li et al., 2025; Yu & Egger, 2021; K. Zhang et al., 2023). The essence of cultural landscapes lies in the historical imprints and symbolic expressions of human activity. They encompass both material entities shaped by human civilization—such as historical architecture, monuments, and traditional villages—and living cultural practices, including festivals, rituals, and artistic performances (Ortanderl & Bausch, 2023; Weng et al., 2021; K. Zhang et al., 2023). The appeal of such landscapes resides in the socio-historical meanings and symbolic values they carry. The visitor experience emphasizes cognitive interpretation and emotional resonance. By understanding the cultural narratives, social functions, and traditional craftsmanship behind these places, visitors gain satisfaction at intellectual, identity-related, or spiritual levels.

In contrast, the defining characteristics of natural landscapes are their primordial nature and ecological integrity, manifested through minimal traces of human intervention (Luo & Chiou, 2021; Stepchenkova & Zhan, 2013). Their constituent elements are primarily non-man-made organic and environmental systems, such as mountains, lakes, seas, forests, and wildlife. The core value of this landscape type lies in providing sensory immersion and restorative experiences (Scannell & Gifford, 2010; Weng et al., 2021). Here, visitors seek detachment from everyday social roles. Through contact with the grand scale of nature, diverse life forms, and serene or sublime environments, they achieve relaxation, aesthetic appreciation, and contemplative engagement.

Thus, cultural and natural landscapes represent two distinct pathways to value generation: the former primarily through the interpretation and connection to cultural meaning, and the latter relying on the sensory attributes and restorative functions of the environment itself. This fundamental distinction provides a critical theoretical context for examining how visual marketing elements—such as the depiction of social cues in imagery—differentially influence tourists’ psychological responses.

## 3. Hypothesis Development

### 3.1. Human Presence, Groundedness, and Destination Attractiveness

Building on the theoretical foundations outlined above, the presence of people in travel photographs may influence viewers’ perceptions of a place through specific psychological processes. Among these, feelings of groundedness may serve as a key mediating mechanism. The presence of people in place photographs can influence groundedness in divergent ways. When depicted individuals embody the cultural meaning of a place (e.g., dancers performing at a traditional festival or artisans practicing local crafts), they may reinforce the symbolic significance of the setting and thereby strengthen viewers’ sense of connection (Z. Y. Lu et al., 2024; Scannell & Gifford, 2010). In many common situations, however, the presence of others disrupts the formation of groundedness. First, from an attentional-allocation perspective, cognitive-psychology research demonstrates that the human visual system exhibits a processing priority for faces and bodily movements (Simhi & Yovel, 2020). When people appear in a photograph, viewers’ gaze is drawn immediately to facial expressions and postures, reducing both the duration and depth of attention devoted to background elements (Y. Li & Xie, 2020). This attentional-dilution effect becomes particularly pronounced when human figures are visually salient—for example, through vivid clothing or dynamic gestures (e.g., Y. W. Li & Wan, 2025; K. Zhang et al., 2023). In place imagery, such attentional shifts can hinder viewers from processing core spatial and cultural cues—such as the architectural layout of traditional villages or the intricate carvings of religious buildings—that foster a sense of place uniqueness, thereby diminishing feelings of groundedness (Birenboim et al., 2013).

Second, from an identity-expression perspective, human presence may also trigger social comparison and identity incongruence, both of which weaken place-based connection. According to self-congruity theory (Sirgy, 1982), individuals are more likely to feel connected to environments that align with their self-concept or desired identity. When depicted individuals appear dissimilar to the viewer in lifestyle, socioeconomic status, or cultural orientation (e.g., affluent tourists portrayed in modest rural villages), such imagery can evoke feelings of “outsiderness” or symbolic exclusion, reducing psychological attachment to the place (Stedman, 2002; Usakli & Baloglu, 2011). Moreover, identity-signaling theory posits that consumption and spatial experiences often function as means of expressing one’s identity to oneself and to others (Berger & Heath, 2007). Human figures that are perceived as incompatible with the viewer’s idealized self-image may therefore contaminate the place’s symbolic meaning, rendering it less suitable as a vehicle for self-expression (Reed et al., 2012). Thus, rather than reinforcing belonging, incongruent human cues can provoke subtle identity threats that distance viewers from the depicted locale.

Building on this reasoning, we propose that although human figures may occasionally convey cultural meaning, their presence more often disrupts viewers’ groundedness. Because groundedness requires sustained cognitive and affective engagement with environmental cues, the visual salience and social signaling of strangers tend to dilute rather than deepen place attachment (Bhattacharjee et al., 2014). Thus, we propose the following hypothesis:

**H1.**  *The presence of other people in place photographs reduces perceived place attractiveness, and this effect is mediated by viewers’ feelings of groundedness.*

### 3.2. The Moderating Role of Landscape Type

In settings rich in cultural symbolism, feelings of groundedness depend on viewers’ ability to process and internalize meaningful cues and to imagine themselves as part of the place’s socio-cultural narrative. Depictions of unrelated others can disrupt this process in two ways. First, because faces and bodies enjoy attentional priority, human figures divert perceptual processing away from core cultural elements—such as ritual practices, architectural details, or historical artifacts—thereby dampening affective resonance (Han & Zhang, 2012; R. Lu et al., 2022). Second, when the depicted individuals appear misaligned with the place identity or the viewer’s self-concept (e.g., casual selfies in sacred spaces), they signal “outsiderness,” undermining imagined membership and, by extension, groundedness (Cohen & Cohen, 2012; C. X. Zhang et al., 2019). Consequently, human presence is expected to reduce perceived attractiveness for cultural landscapes by weakening viewers’ feelings of groundedness.

By contrast, in nature-oriented contexts, groundedness relies less on socio-symbolic interpretation and more on direct sensory immersion in environmental features (F. S. Li et al., 2025). Characteristics such as vastness, fractal patterns, and dynamic light and shadow produce restorative effects that dominate perceptual processing (Kaplan, 1995; Jiang et al., 2015). These qualities capture attention, reduce stress, and diminish the salience of social cues, rendering human presence peripheral rather than disruptive. Moreover, awe-inspiring vistas can evoke a “small self” that reduces social comparison (Piff et al., 2015), further buffering against potential interference. Because human figures contribute little diagnostic value to the appeal of natural landscapes, the attentional costs and potential benefits of their inclusion tend to offset one another, resulting in negligible effects. Hence, in natural settings, human presence is unlikely to meaningfully alter feelings of groundedness or place attractiveness.

Taken together, these arguments suggest that landscape type serves as a boundary condition for the effects of human presence in place photographs. Whereas natural landscapes buffer against distraction, cultural landscapes are more vulnerable to disruption, making viewers less likely to experience groundedness when others are depicted. Thus, we propose the following hypothesis:

**H2.**  *Landscape type moderates the effect of human presence in place photographs on perceived place attractiveness. In cultural (vs. natural) landscapes, the presence of people reduces groundedness and, in turn, place attractiveness.*

**Figure 1:**
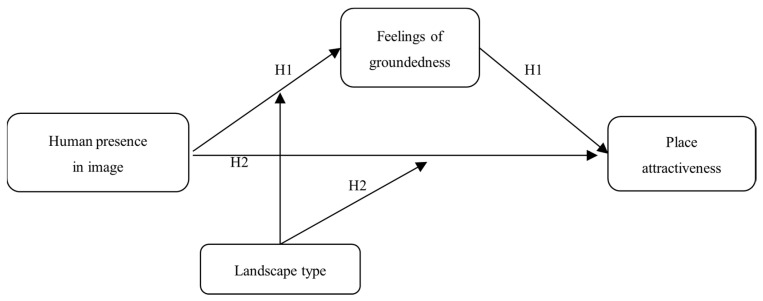
Theoretical Framework.

Figure 1 presents the conceptual framework of this research. The independent variable is human presence in place photographs; the dependent variable is perceived place attractiveness; the mediating variable is feelings of groundedness; and the moderating variable is landscape type, which moderates the effect of human presence on feelings of groundedness. We conducted four studies—one field study and three controlled laboratory experiments—to empirically test this framework.

## 4. Overview of Studies

Across four studies, we test this theorization using a multi-method approach that integrates field and experimental designs. Study 1 establishes the main effect of human presence on engagement-based behavioral measures in a real-world marketing context. Study 2 replicates the effect under controlled conditions and identifies groundedness as the mediating mechanism. Study 3 introduces landscape type (cultural or natural landscapes) as a boundary condition, showing that the negative impact of human presence emerges in cultural but not natural landscapes. Study 4 provides full moderated mediation evidence and rules out potential confounds such as psychological ownership. Together, these studies advance understanding of how visual social cues shape consumer responses to place imagery.

## 5. Methods

### 5.1. Study 1

To provide initial evidence for the effect of human presence in place imagery, we conducted a field experiment on TikTok to examine whether human presence influences viewers’ engagement with destination-related content.

#### 5.1.1. Design and Procedure

Using an A/B testing design, we created two promotional videos that were identical in length, background music, and presentation format but differed in the inclusion of people. Each video comprised four destination photographs—two depicting natural landscapes and two depicting cultural landmarks. In one condition, all four images excluded people, whereas in the other condition, each image featured a visible human figure within the same scenes. Consistent with prior research (e.g., Y. W. Li & Wan, 2025; F. S. Li et al., 2025), we used images depicting people from behind to eliminate the potential confounding effect of facial attractiveness (see Appendix A for the stimuli).

Both videos were posted simultaneously on TikTok and received at least 5000 views each, supported by TikTok’s official promotion algorithm for 24 h. We confirmed that the audiences of the two videos were mutually exclusive; each user was exposed to only one version. Following prior social media marketing research (Lee & Hong, 2016), we used ‘likes’ as the main indicator of engagement. Additionally, we tracked the two-second drop-off rate and completion rate, both key measures of video effectiveness on TikTok (Evans, 2024).

#### 5.1.2. Results

After 24 h, the no-human video received 7550 views, 527 completions, 890 two-second drop-offs, and 109 likes. The human-present version received 6589 views, 211 completions, 1581 drop-offs, and 48 likes. Chi-square analyses revealed that the no-human version significantly outperformed the human-present version across all metrics: completion rate increased by 118% (6.98% vs. 3.20%; Odds Ratio = 2.27, *p* < 0.001), two-second bounce rate decreased by 51% (11.79% vs. 23.99%; Odds Ratio = 0.42, *p* < 0.001), and like rate nearly doubled (1.44% vs. 0.73%; Odds Ratio = 1.98, *p* < 0.001).

#### 5.1.3. Discussion

These results demonstrate that destination videos with people generate lower engagement across behavioral measures. Notably, nearly one-quarter of viewers exited the video with a human figure within two seconds, whereas the no-human version retained almost twice as many viewers, suggesting that human presence may immediately divert attention away from the landscape and weaken immersive processing.

Study 1 thus provides field-based behavioral evidence that excluding people from destination imagery enhances engagement. However, this study does not clarify why human presence reduces engagement, and causal inference remains limited due to TikTok’s opaque recommendation algorithm. Study 2 addresses these limitations through controlled experimentation.

### 5.2. Study 2

The purpose of study 2 was twofold. First, it employed a controlled experiment to more rigorously test whether human presence influences perceived place attractiveness. Second, it examined the underlying psychological mechanism driving this effect. We propose that feelings of groundedness serve as the key mediating mechanism. In addition, we measured participants’ psychological ownership of the place after viewing the photographs, as the presence of people might evoke a sense that the place has already been “claimed” or “occupied” by others. Such perceived ownership could, in turn, help explain why destinations depicted with people may appear less attractive than those without human presence (Z. Y. Lu et al., 2024).

#### 5.2.1. Design and Procedure

A single-factor between-subjects design (human presence: present vs. absent) was used. Four hundred participants (*M*_age = 29.35; 29.8% male) were recruited via the Credamo platform, a Chinese online participant pool similar to MTurk (Chen et al., 2024). Participants imagined searching for a leisure destination for the upcoming weekend and viewed one photograph ostensibly posted by a travel blogger. The two versions differed only in the inclusion of a person (see Appendix B).

After viewing the image, participants evaluated the destination and the photograph (Y. W. Li & Wan, 2025; F. S. Li et al., 2025). Destination evaluation was measured with three items adopted from previous studies (after viewing the photo, I think this place is: very unattractive/very attractive; very unaesthetic/very aesthetic; not at all worth visiting/very much worth visiting; α = 0.87). Photo evaluation was measured with parallel three items (after viewing the photo, I think this photo is: very unattractive/very attractive; very unaesthetic/very aesthetic; very visually unappealing/very visually appealing; α = 0.90). Drawing on existing literature (Eichinger et al., 2022), feelings of groundedness were measured using three seven-point Likert items: “This photo makes me feel deeply rooted in this place”, “This photo gives me a sense of belonging to this place”, and “This photo makes me want to feel connected to this place” (1 = strongly disagree, 7 = strongly agree; Eichinger et al., 2022; α = 0.88).

To ensure the validity of our focal construct—feelings of groundedness—we adopted the well-established scale developed by Eichinger et al. (2022), which demonstrates strong content and construct validity in capturing individuals’ sense of connection and rootedness in a place. Across our studies, the scale exhibited high internal consistency (Study 2: α = 0.88; Study 4: α = 0.87), indicating reliable measurement in the context of place imagery evaluation. The place attractiveness measure, adapted from prior tourism marketing research (e.g., Y. W. Li & Wan, 2025), also showed excellent reliability across all studies (α ranging from 0.87 to 0.90), supporting its suitability as the dependent variable.

To rule out the alternative explanation of psychological ownership, we measured participants’ perceived sense of ownership toward the place using two seven-point Likert items adapted from Z. Y. Lu et al. (2024): “This photo makes me feel that the place is already occupied by others”, “This photo makes me feel that the place already belongs to someone else” (1 = strongly disagree, 7 = strongly agree: r = 0.83). Finally, participants reported demographic information (i.e., age and gender).

#### 5.2.2. Results

A one-way ANOVA revealed a significant effect of human presence on place evaluation. Participants rated the place more favorably when the photograph did not include people (*M* = 5.64, SD = 1.14) than when it did (*M* = 5.29, SD = 1.28), F (1, 398) = 8.04, *p* = 0.005. A similar effect emerged for photo evaluation, with higher ratings in the no-human condition (*M* = 5.54, SD = 1.22) than in the human-present condition (*M* = 5.29, SD = 1.27; F (1, 398) = 3.98, *p* = 0.047). These results provide convergent support for H1, demonstrating that human presence reduces both perceived photo and place attractiveness.

Consistent with our predictions, the presence of people in the photo led to a reduction in participants’ perceptions of groundedness toward the place (*M*_present = 4.85, SD = 1.38 vs. *M*_absent = 5.14, SD = 1.19; F (1, 398) = 5.03, *p* = 0.025). However, human presence did not affect participants’ perceived psychological ownership of the place (*M*_present = 2.88, SD = 1.44 vs. *M*_absent = 2.89, SD = 1.40; F (1, 398) < 1, *p* = 0.93).

A mediation analysis (PROCESS Model 4, 10,000 bootstraps; Hayes, 2017) revealed that feelings of groundedness mediated the effect of human presence on photo attractiveness (β = −0.20, SE = 0.09, 95% CI [−0.3854, −0.0302]) and also mediated the effect of human presence on place attractiveness (β = −0.20, SE = 0.09, 95% CI [−0.3784, −0.0234]). Furthermore, a sequential mediation analysis (PROCESS Model 6, 10,000 bootstraps; Hayes, 2017) was conducted with human presence as the independent variable, place attractiveness as the dependent variable, and groundedness feelings and photo attractiveness as sequential mediators. The results indicated a significant indirect effect (β = −0.13, SE = 0.06, 95% CI [−0.2624, −0.0149]), suggesting that groundedness feelings and photo attractiveness sequentially mediated the effect of human presence on place attractiveness.

To rule out the alternative explanation of psychological ownership, we conducted parallel mediation analyses using psychological ownership as the mediator. The results showed no significant mediation effect, as the 95% confidence intervals included zero (place attractiveness: β = 0.004, SE = 0.04, 95% CI [−0.0827, 0.0888]; photo attractiveness: β = 0.004, SE = 0.04, 95% CI [−0.0839, 0.0895]).

#### 5.2.3. Discussion

The results of study 2 confirm that human presence in place photographs lowers evaluations relative to images without people. Whereas study 1 demonstrated this effect in a field setting using behavioral metrics, study 2 replicates the finding in a controlled experimental context with direct attitudinal measures.

Moreover, the findings reveal that feelings of groundedness mediate the effect of human presence on place attractiveness. Specifically, when a place image contains other people, participants report weaker groundedness feelings—a diminished sense of psychological rootedness and connection to the place—which, in turn, lowers their evaluations of the place. At the same time, this study rules out psychological ownership as an alternative explanation. Prior research suggests that the presence of others in a place image may lead individuals to perceive that the place has already been “claimed” by others, thereby reducing their own perceived ownership and subsequent evaluations (Z. Y. Lu et al., 2024). However, our results indicate that this account does not hold in the current context.

We further examined whether reduced groundedness feelings may hinder the development of psychological ownership, thereby indirectly lowering place evaluations. A sequential mediation analysis (PROCESS Model 6, 10,000 bootstraps; Hayes, 2017) with human presence as the independent variable, place attractiveness as the dependent variable, and feelings of groundedness and psychological ownership as sequential mediators revealed a significant indirect effect (β = −0.01, SE = 0.007, 95% CI [−0.0272, −0.0012]). Taken together, given that psychological ownership alone did not mediate the effect of human presence on place attractiveness, whereas feelings of groundedness did, these findings suggest that groundedness feelings represent a deeper and more fundamental psychological mechanism explaining why the presence of people in destination images attenuates place evaluations.

### 5.3. Study 3

Building upon the findings of study 2, study 3 aimed to address a key unresolved question: Does the effect of human presence depend on contextual factors, such as the type of landscape depicted? To this end, we examined whether the landscape type (cultural vs. natural) moderates the impact of human presence on perceived place attractiveness.

#### 5.3.1. Design and Procedure

Study 3 employed a 2 (human presence: present vs. absent) × 2 (landscape type: cultural vs. natural) between-subjects experimental design. We recruited 394 participants (mean age = 30.14 years; 28.4% male) via the Credamo online platform and randomly assigned them to one of the four conditions. The procedure closely mirrored that of study 2. Participants viewed a single place photograph and then evaluated the place attractiveness. The cultural landscape image was identical to that used in study 2, whereas the natural landscape image depicted an uninhabited natural scene (see Appendix C). After viewing the image, participants completed the same three-item measure of place attractiveness used previously (α = 0.89). Finally, they reported demographic information (i.e., age and gender).

#### 5.3.2. Results

**Figure 2:**
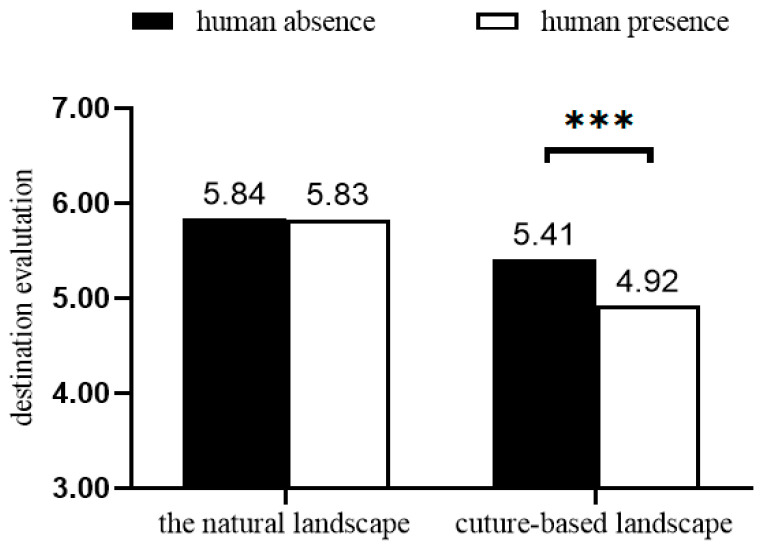
The moderating role of landscape type (Study 3). *** *p* < 0.001.

To test the moderating effect of landscape type on the relationship between human presence and perceived attractiveness, we conducted a two-way ANOVA. Results indicated a significant interaction between human presence and landscape type on place attractiveness (F (1, 390) = 4.08, *p* = 0.044; see Figure 2). Follow-up simple effects analyses showed that when the image depicted a cultural landscape, the presence of people reduced perceived place attractiveness (*M*_present = 4.92, SD = 1.52 vs. *M*_absent = 5.41, SD = 1.05; F (1, 390) = 8.62, *p* = 0.0035). In contrast, when the image depicted a natural landscape, human presence had no effect on attractiveness evaluations (*M*_present = 5.83, SD = 0.98 vs. *M*_absent = 5.84, SD = 1.00; F (1, 390) < 1, *p* = 0.919).

#### 5.3.3. Discussion

Study 3 confirmed that landscape type moderates the effect of human presence on place attractiveness. Consistent with hypothesis 2, the negative influence of human presence emerged only for cultural landscapes but not for natural landscapes.

We propose that this pattern reflects the differing psychological engagement processes evoked by the two landscape types. Experiencing a cultural landscape typically requires individuals to form a deeper connection with its people, traditions, and historical meanings. The visible presence of others in such images may disrupt this potential for immersion, thereby diminishing feelings of groundedness and, in turn, reducing place evaluations. In contrast, natural landscapes evoke appreciation that is more aesthetic and contemplative, and thus less susceptible to social cues such as human presence. To further validate this interpretation, study 4 was designed to directly measure feelings of groundedness as a mediating process and provide a complete test of hypothesis 2.

### 5.4. Study 4

The primary purpose of study 4 was twofold. First, it sought to provide a full test of hypothesis 2 by replicating the 2 (human presence: present vs. absent) × 2 (landscape type: cultural vs. natural) between-subjects design used in study 3, while additionally measuring the proposed mediator—feelings of groundedness. Second, given that individuals may differ in how they perceive and react to others’ presence, we also introduced self-construal as an exploratory moderator. Prior literature suggests that individuals with an interdependent (vs. independent) self-construal tend to form closer interpersonal bonds and display greater trust toward others (Cross et al., 2000; Markus & Kitayama, 1991). Accordingly, we explored whether self-construal moderates the effect of human presence on place evaluations.

#### 5.4.1. Design and Procedure

We recruited 300 participants via the Credamo platform (mean age = 30.96 years, 29.3% male) and randomly assigned them to one of four experimental conditions in a 2 × 2 between-subjects design. The procedure and stimuli closely followed those of study 3. Participants imagined planning a weekend getaway and viewed one place photograph—either a cultural or natural landscape, each depicted with or without people.

After viewing the photograph, participants rated place attractiveness (α = 0.90), followed by feelings of groundedness using the same three items as in study 2 (α = 0.87). Subsequently, they completed the self-construal scale (Chen & Huang, 2016; Gudykunst et al., 1996), consisting of six interdependent items (α = 0.82) and six independent items (α = 0.73). We constructed a self-construal index as follows: (interdependent − independent)/(interdependent + independent) (see Escalas & Bettman, 2005; Winterich & Barone, 2011), with higher scores reflecting a greater interdependent tendency. Finally, demographic information was collected (i.e., gender and age).

#### 5.4.2. Results

A 2 × 2 ANOVA revealed a significant interaction between human presence and landscape type on place evaluations (F (1, 296) = 5.00, *p* = 0.026). Simple effect analyses showed that when the place was a cultural landscape, human presence significantly reduced attractiveness perceptions (*M*_present = 5.00, SD = 1.47 vs. *M*_absent = 5.66, SD = 1.18; F (1, 296) = 12.01, *p* = 0.0006). However, for natural landscapes, this effect disappeared (*M*_present = 5.65, SD = 1.03 vs. *M*_absent = 5.71, SD = 0.89; F < 1, *p* = 0.761).

**Figure 3:**
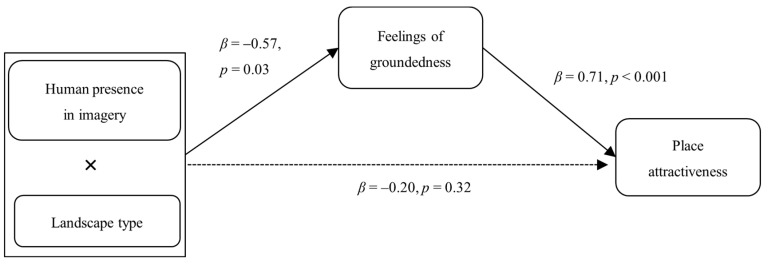
The moderated mediation model of Study 4.

To test whether feelings of groundedness mediated the interaction between human presence and landscape type, we conducted a moderated mediation analysis using PROCESS Model 8 (Hayes, 2017). Destination evaluation served as the dependent variable, human presence (0 = absent, 1 = present) as the independent variable, feelings of groundedness as the mediator, and landscape type (0 = natural, 1 = cultural) as the moderator. Results revealed a significant overall moderated mediation effect (β= −0.40, SE = 0.19, 95% CI [−0.7823, −0.0374]). The indirect effect of human presence on place evaluation through groundedness feelings was significant only in cultural landscapes (β= −0.57, SE = 0.15, 95% CI [−0.8776, −0.2589]), but not in natural landscapes (β= −0.17, SE = 0.12, 95% CI [−0.4065, 0.0597]). These results support hypothesis 2, confirming that feelings of groundedness mediate the human presence effect only when the destination is cultural (see Figure 3).

We next examined whether self-construal moderated the effects of human presence and landscape type. Using PROCESS Model 3 (Hayes, 2017), we tested the three-way interaction among human presence, landscape type, and self-construal on place evaluations. The three-way interaction was nonsignificant (β= −0.45, SE = 2.50, *p* = 0.86), and self-construal did not interact with either human presence or landscape type individually (*ps* > 0.50). We also re-ran the 2 × 2 ANCOVA controlling for self-construal, and the human presence × landscape type interaction remained significant (F (1, 295) = 5.03, *p* = 0.026). Simple effects mirrored those of the original ANOVA. These findings indicate that self-construal does not moderate the human presence effect, suggesting that the phenomenon operates robustly across self-construal orientations.

#### 5.4.3. Discussion

Study 4 provides a full test of the proposed moderated mediation model, confirming that human presence diminishes perceived attractiveness of cultural—but not natural—landscapes through reduced feelings of groundedness. The absence of moderation by self-construal suggests that this process operates robustly across individual differences in self-view. Overall, study 4 consolidates the evidence that human presence can interrupt the psychological connection between viewers and culturally rich environments, thereby weakening place appeal.

## 6. General Discussion

### 6.1. Summary

Across four studies, the present research yields a consistent empirical pattern regarding the role of human presence in place imagery, providing strong support for both H1 and H2 and helping to resolve prior mixed findings in the literature. Specifically, depicting people in place photographs tends to reduce perceived place attractiveness, and this effect operates through a previously underexplored psychological mechanism—groundedness. The presence of others weakens viewers’ sense of psychological rootedness and connection to the depicted place, which in turn diminishes evaluative responses.

Importantly, the findings further clarify the contextual boundary conditions underlying this effect. For cultural landscapes, where place evaluations rely heavily on symbolic meaning and socio-cultural interpretation, human presence reliably undermines groundedness and perceived attractiveness. In contrast, for natural landscapes, where evaluations are less dependent on such symbolic engagement, the presence of people does not meaningfully influence either groundedness or place evaluations. Taken together, these results offer an integrative account of when and why human presence in place imagery can backfire by specifying both the underlying psychological process and the conditions under which this effect emerges.

### 6.2. Theoretical Contributions

This research offers several key theoretical contributions. First, it provides an integrative account of the mixed evidence surrounding the effects of human presence in place imagery. Prior work has shown that including people can facilitate mental simulation and help potential tourists imagine themselves in a scene, thereby enhancing perceived attractiveness and visit intentions (Y. W. Li & Wan, 2025; K. Zhang et al., 2023). Yet other studies reveal that human presence can evoke perceptions that the place “belongs to others,” reducing liking and preference for the venue (Z. Y. Lu et al., 2024). By systematically comparing the presence versus absence of people, this research offers an integrative framework that reconciles these divergent findings. Specifically, we demonstrate that, although human figures are often assumed to increase relatability and appeal, their inclusion can under many conditions undermine place attractiveness. In doing so, we move beyond piecemeal explanations to clarify the contextual contingencies under which human presence in promotional imagery backfires.

Second, this research advances theory by introducing and empirically validating the construct of groundedness (Bruckberger et al., 2023; Eichinger et al., 2022; Guo et al., 2024; Y. Xie & Desouza, 2025). Groundedness captures individuals’ cognitive and emotional anchoring to a place—reflecting their sense of belonging, stability, and security in relation to a place. Building on prior conceptualizations (Eichinger et al., 2022) and subsequent scale development in cultural tourism contexts (Guo et al., 2024), as well as work on retail experiences (Bruckberger et al., 2023), this research extends the construct to the domain of visual marketing. By showing that diminished feelings of groundedness mediate the negative effect of human presence on perceived attractiveness, we establish groundedness as a novel and central psychological mechanism explaining viewers’ responses to place imagery. Importantly, our findings suggest that existing accounts—such as psychological ownership (Z. Y. Lu et al., 2024)—cannot fully explain why human presence sometimes erodes rather than enhances place appeal. Thus, this research enriches the theoretical toolkit of consumer and tourism psychology by illuminating an emotional process that underlies both connection to and detachment from place.

Third, this research identifies landscape type as a critical moderator that qualifies the human-presence effect. While prior studies have focused on the identity or activities of depicted individuals (F. S. Li et al., 2025; Z. Y. Lu et al., 2024), we demonstrate that the broader environmental context fundamentally shapes consumer responses. Human presence diminishes groundedness and attractiveness in cultural landscapes, where meaning depends on symbolic coherence and heritage continuity. In contrast, in natural landscapes, restorative perceptual processing and attentional absorption in the environment render human presence largely inconsequential. By establishing landscape type as a boundary condition, this research not only clarifies when the negative effects of human presence occur but also provides a more nuanced theoretical framework for understanding how environmental contexts moderate the impact of visual social cues on consumer evaluations.

### 6.3. Practical Implications

Beyond its theoretical contributions, this research provides actionable insights for tourism marketers and visual content designers. The findings suggest that the inclusion of people in place imagery should not be treated as an inherently persuasive strategy. While marketers often assume that showing human figures enhances relatability and social warmth, our results indicate that such imagery can inadvertently diminish perceived attractiveness—particularly in cultural contexts where the symbolic and historical meanings of the setting are central to its appeal.

For cultural destinations, visual communication should prioritize environmental and architectural cues that evoke authenticity, heritage continuity, and spatial harmony rather than crowding the scene with unrelated individuals. Images that highlight craftsmanship, rituals, or the aesthetic qualities of the environment itself are more likely to foster feelings of groundedness and emotional connection. Marketers should therefore adopt a “less-is-more” approach, emphasizing the intrinsic character of the place over generic depictions of tourists.

For natural destinations, human presence appears less detrimental, as such environments draw appeal primarily from restorative and sensory immersion. Here, selectively including small, contextually appropriate figures (e.g., a lone hiker or diver) may even help convey scale and accessibility without distracting from the environment’s core visual impact.

Moreover, social media and influencer campaigns should carefully calibrate the balance between personal relatability and spatial authenticity. When promoting cultural heritage sites or sacred locations, user-generated content guidelines might encourage imagery that respects the site’s symbolic integrity. Destination management organizations could further leverage groundedness-based framing—emphasizing connection, belonging, and stability—to enhance emotional engagement in their visual storytelling.

Finally, these insights extend beyond tourism. For marketers in retail, real estate, or hospitality, our findings highlight the broader principle that visual social cues can both humanize and disrupt consumers’ sense of psychological connection with a place (Z. Y. Lu et al., 2024). Effectively managing the presence and contextual fit of people in visual materials can therefore serve as a subtle yet powerful lever for shaping consumer experience and persuasion (F. S. Li et al., 2025; Y. W. Li & Wan, 2025).

### 6.4. Limitations and Future Research

While this research provides robust evidence for the role of human presence and groundedness in shaping responses to place imagery, several limitations offer promising avenues for future research.

First, our studies focused primarily on still photographs as the visual medium (except study 1). Yet, in digital tourism marketing, short-form videos and immersive media (e.g., VR, AR) are becoming dominant (Liu et al., 2018). Future work could examine whether dynamic motion cues, background music, or narrative voiceovers amplify or mitigate the effects of human presence on feelings of groundedness. Extending this inquiry to multi-sensory contexts would enrich understanding of how groundedness operates in more realistic and interactive environments.

Second, the current research examined human presence as a binary factor—present versus absent. In practice, social cues vary not only in quantity but also in characteristics such as identity, behavior, emotional expression, and cultural fit (F. S. Li et al., 2025; Z. Y. Lu et al., 2024). Future research could explore whether the effects of human presence depend on who is depicted (e.g., locals vs. tourists, same-culture vs. cross-culture figures) or what they are doing (e.g., performing rituals vs. leisure activities). Such extensions could clarify whether certain portrayals strengthen groundedness by reinforcing cultural coherence rather than undermining it.

Third, future work could explore boundary mechanisms beyond landscape type. While we identified cultural versus natural settings as a key moderator, other contextual factors—such as crowd density, image framing, or place familiarity—may also shape the degree to which human presence disrupts or enhances groundedness. Moreover, longitudinal designs could investigate how repeated exposure to human-inclusive imagery influences memory, affective forecasting, and actual travel behavior over time.

## 7. Conclusions

This research demonstrates that the presence of people in destination imagery does not uniformly enhance place attractiveness; instead, its effects depend on how visual social cues shape viewers’ psychological connection to the place. By showing that human presence can weaken feelings of groundedness—particularly in contexts where symbolic meaning and cultural interpretation are central—this study highlights groundedness as a foundational mechanism in visual place evaluation. More broadly, the findings underscore the importance of aligning visual design choices with the experiential essence of the destination, suggesting that effective place imagery should be guided not by the mere inclusion of social elements, but by a nuanced understanding of how such elements influence perceived connection and appeal.

## Data Availability

The data presented in this study are available on request from the corresponding author. The data are not publicly available due to participant privacy restrictions.

## Appendix B. Stimuli in Study 2

The experimental stimuli consisted of the third set of images (with and without people) from Study 1.

## Appendix C. Stimuli in Study 3

The natural and cultural landscape materials were from Sets 2 and 3 of Study 1, respectively.
